# Prevalence and significance of a canine bocavirus-2 outbreak in a cohort of military dogs in Austria

**DOI:** 10.3389/fvets.2024.1461136

**Published:** 2024-09-05

**Authors:** P. G. Doulidis, R. Reisner, A. Auer, Katharina Dimmel, Thomas Lammer, F. Künzel

**Affiliations:** ^1^Clinical Unit of Internal Medicine Small Animals, Department for Companion Animals and Horses, University of Veterinary Medicine, Vienna, Austria; ^2^Department of Pathobiology, Institute of Virology, University of Veterinary Medicine Vienna, Vienna, Austria; ^3^Militärhundezentrum Kaisersteinbruch, Kaisersteinbruch, Austria

**Keywords:** canine, bocavirus, *Parvoviridae* infections, PCR, outbreak

## Abstract

**Introduction:**

Bocaviruses are single-stranded DNA viruses from the *Parvoviridae* family, which have been minimally discussed in veterinary literature and are considered potentially pathogenic. Due to the recurring illness among young dogs in a closed cohort of military dogs in Austria, we assessed the prevalence, possible disease manifestation and outcome of CBoV-2 infection in this cohort.

**Materials and methods:**

This led to a comprehensive study that not only analyzed past cases but also performed prospective screening PCR tests to identify CBoV-2 positive dogs within this specific dog population. Pharyngeal and rectal swabs were taken. In addition, a control group (*n* = 20) of clinically healthy client-owned dogs was sampled. A total of 190 samples were taken and tested for the presence of CBoV-2 specific nucleic acid using screening PCR. In addition to the primers used for routine diagnostics, two other primer pairs were used to verify questionable results. The retrospective part of the study includes a total of 13 military dogs that had previously shown suspected clinical signs.

**Results:**

At the time of the first examination within the prospective part of the study, CBoV-2 was detected in 31% (12/39) of the dogs. During the second examination, 2% (1/47) tested positive, while all PCR testing in the control group (*n* = 20) was negative in all cases. The retrospective evaluation of the 13 cases revealed a total of six animals tested positive for CBoV-2 via screening PCR. All puppies suffered from skin lesions (papules, vesicles, or pustules). Other clinical signs included diarrhea (83%), vomiting (77%), respiratory (15%), and neurological (8%) signs.

**Discussion:**

According to the study there are certain indications that CBoV-2 shares similarities with CPV-2 infection but also exhibits critical differences, making their differentiation essential for patient management, outcomes, and prevention strategies.

## Introduction

Bocaviruses, are single-stranded DNA viruses from the *Parvoviridae* family that have been identified in a wide range of mammalian hosts, including primates, pigs, cattle, chiropterans, dogs, cats and rodents, and are predominantly associated with pathologies of the gastrointestinal or respiratory systems (1, 2). The genus *Bocaparvovirus* derives its nomenclature from two originally designated viruses: bovine parvovirus (BPV) and canine minute virus (CnMV) (3). To date, the taxonomic classification of the genus *Bocaparvovirus* encompasses 31 recognized species, with three having been documented in canine hosts. Among these, CnMV (species *Bocaparvovirus carnivoran1*), identified during the late 1960s, continues to elude comprehensive understanding regarding its pathogenicity. Specifically, it is linked to severe neonatal respiratory afflictions and enteritis in juvenile canines, while infections in adult canids typically remain latent. Even with the phylogenetic affinity within the same subfamily, CnMV exhibits notable evolutionary divergence from other classified canine bocaviruses (4). In addition to CnMV, two other *bocaparvoviruses*, namely canine bocavirus 2 (CBoV-2) and canine bocavirus 3 (CBoV-3), have been identified in canine hosts (4, 5).

The discovery of CBoV-2 in 2012 marked its initial detection in canine populations, prompting speculation regarding its pathogenic potential. Its association has been noted in canines presenting with respiratory and gastrointestinal disorders; however, conclusive evidence attributing it as the sole etiological agent for observed clinical manifestations remains elusive (4, 6). In spite of it being a DNA virus, CBoV-2 exhibits notable genomic recombination, contributing to its diverse genotypic landscape. Nevertheless, the precise impact of this genomic diversity on its pathogenicity or clinical phenotypes remains indeterminate (6). In 2013, the identification of canine bocavirus 3 (CBoV-3) in the liver of a canine specimen, alongside concurrent co-infection with a novel canine circovirus was reported and phylogenetic analyses unveiled a distinct evolutionary trajectory, separating CBoV-3 from both CnMV and CBoV-2 (5). Currently, there is a lack of documented cases of CBoV-3 in the scientific literature.

CBoV-2 has been identified through molecular biological techniques in canine populations across Germany, South Korea, the People’s Republic of China, Thailand and Hong Kong (7, 8). Notably, recent research has unveiled the presence of CBoV-2 with a prevalence rate of 5% in wild carnivores, specifically gray wolves inhabiting northern Canada (9). Moreover, a subsequent investigation by Canuti et al. examining the prevalence of various parvoviruses [canine parvovirus 2 (CPV-2), CBoV-2, and CnMV] among diverse canine species, including coyotes, foxes, and domestic dogs, revealed CBoV-2 in 10% of canine fecal samples. However, attempts to isolate CBoV-2 from coyotes and foxes, encompassing fecal, splenic, and lymphatic tissue samples, yielded negative outcomes (10). Broadly, the clinical severity associated with *Bocaparvovirus* infection appears to manifest more severely in juvenile cohorts compared to their adult counterparts (2). As for CBoV-3, its singular detection thus far has been confined to the hepatic tissue of a canine specimen from the United States (5).

The pathogenicity, mode of infection, and cell tropism of CBoV-2 remain unclear to date. Attempts to establish cell cultures for virus isolation and propagation have thus far been unsuccessful. Additionally, suitable *in vivo* models are currently unavailable (11). Consequently, the Koch’s postulates have not been fulfilled due to the lack of data, preventing the establishment of a causal relationship between the presence of the virus in affected organs of diseased individuals and the observed clinical signs. Furthermore, there is ongoing debate regarding whether bocaviruses act as primary pathogens or exhibit synergistic effects, as they are often detected in the context of co-infections (12). Originally, CBoV-2 was detected in respiratory tissue samples from deceased dogs during a metagenomic study (4). The infected dogs, sourced from a shelter, were associated with an elevated prevalence compared to our control population. Other pathogenic respiratory viruses were not tested and could not be excluded as causal agents. The exact cause of death in these animals was not described, leading to ongoing discussions regarding the potential role of CBoV-2 as a causal pathogen in canine respiratory diseases. In another study focusing on dogs presenting notable dyspnea and hemoptysis, a potential association between CBoV-2 detection and concurrent intestinal lesions was suggested. Although initial assumptions pointed to respiratory failure in these dogs, histopathological examinations revealed no significant changes in the lungs or trachea. However, CBoV-2 was detected by PCR in the lung, tracheal lymph nodes, liver, and intestine, raising questions about its potential involvement in respiratory and gastrointestinal pathologies (6).

The aim of this study was to evaluate the frequency of CBoV-2 in our study population, to describe the clinical signs and disease course in CBoV-2 positive dogs and their littermates and to determine the potential spread of the virus within a well-defined dog population. Herein, we present the results of a prospective and a retrospective study.

## Materials and methods

### Prospective study

For the prospective part of this study, a total of 52 dogs from a military dog center in Austria with suspected bocavirus infection were included. Rectal, throat, and where possible lesion swabs were obtained from the animals and subsequently examined for CBoV-2 using conventional PCR. When possible, a histopathological examination and PCR testing of blood, urine, and various organs were performed. Before sampling, approval from the Ethics and Animal Welfare Committee of the University of Veterinary Medicine Vienna was obtained (ETK No. 019/02/2021). All welfare and ethic standards were met for this cohort and are under control of the government. At the time of sample collection, the breed, age, and gender were documented on-site, and a brief medical history of each sampled dog was obtained. An overview of the data is provided in Table 1. The population consisted of Rottweilers bred within the military dog center, working dogs, and private dogs of the staff members, who were present on the premises of the military dog center almost on a daily basis. Additionally, military dogs of external personnel present on the day of the second sampling session were included in this study to assess potential spread of CBoV-2. Furthermore, a control group was established to confirm the hypothesis that the military dog center is an endemically affected population. Data from these animals were documented by one of the authors using a questionnaire. Samples from these dogs were collected by the owners in the form of fecal swabs and then subjected to screening for CBoV-2 using PCR at the Institute of Virology, University of Veterinary Medicine Vienna. An overview of the control group (breed, gender, age) is provided in Table 2. The sample collection was conducted at two time points, approximately 2 months apart (June 29, 2021, and August 30, 2021). Rectal and oropharyngeal swabs were taken from all animals on-site. Flocked dry swabs (FLOQSwabs™, Copan Italia S.p.A., Brescia, Italy) were used for sample collection. Documentation of each dog’s data was performed on-site (Table 1). For the collection of both swabs, the animals were briefly restrained by their respective handlers and/or present staff members of the military dog center. The swabs were taken by one of the authors of the present study or the attending veterinarians of the Military Dog Center. Oropharyngeal swabs were taken with the animals in a sitting position. Opening of the mouth was performed by the handler to minimize the stress of the animal during sampling. The swab was taken by rotating movements in the tonsils. Rectal swabs were taken immediately afterward with the dog standing; the swabs were inserted rectally for a few seconds with rotating movements approximately 1–2 cm into the rectum. All samples were immediately labeled with the animal’s name and the site of sample collection, then cooled using ice packs. Subsequently, they were promptly transported to the Institute of Virology at the University of Veterinary Medicine Vienna and stored at −80°C for further processing.

**Table 1:** Overview of the data of the sampled population.

No.	Breed	Gender	Age	First sampling (June)	Second sampling (August)
1	Malinois	Male	Unknown	X	✓
2	Malinois	Female	Unknown	✓	✓
3	Malinois	Male	1 year 9 months	✓	✓
4	Rottweiler	Male	4 years 2 months	✓	✓
5	Labrador	Male	8 years 10 months	✓	✓
6	GSD	Male	1 year 5 months	✓	✓
7	Labrador	Male	1 year 6 months	✓	X
8	Labrador	Male	1 year 4 months	X	✓
9	Labrador	Male	1 year 4 months	X	✓
10	GSD	Male	3 years 7 months	X	✓
11	Rottweiler	Male	Unknown	✓	✓
12	GSD	Male	2 years 9 months	✓	✓
13	Labrador	Male	1 year 6 months	✓	X
14	Rottweiler	Male	3 years 1 months	✓	✓
15	Malinois	Female	7 years 4 months	✓	✓
16	Labrador	Male	1 year 6 months	✓	✓
17	Rottweiler	Female	3 years 1 months	✓	✓
18	Labrador	Male	1 year 6 months	✓	✓
19	Tervueren	Female	8 years 1 month	✓	✓
20	Malinois	Male	Unknown	X	✓
21	Malinois	Female	1 year 5 months	X	✓
22	Rottweiler	Female	2 years 9 months	✓	✓
23	Malinois	Male	1 year 5 months	X	✓
24	Rottweiler	Male	2 years 9 months	✓	✓
25	Rottweiler	Male	Unknown	X	✓
26	Malinois	Female	1 year 5 months	X	✓
27	Rottweiler	Male	2 years 9 months	✓	✓
28	Rottweiler	Male	2 years 9 months	✓	✓
29	Rottweiler	Male	2 years	✓	✓
30	Rottweiler	Female	2 years	✓	✓
31	Rottweiler	Female	2 years	✓	✓
32	Rottweiler	Female	6 years	✓	X
33	Labrador	Male	10 years 5 months	✓	✓
34	Rottweiler	Female	Unknown	X	✓
35	Rottweiler	Female	12 years 3 months	✓	✓
36	Malinois	Male	10 years 9 months	✓	✓
37	Malinois	Male	Unknown	X	✓
38	Rottweiler	Female	6 years 2 months	✓	✓
39	Malinois	Male	4 years 4 months	✓	✓
40	Labrador	Female	7 years 8 months	✓	✓
41	Labrador	Male	7 years 8 months	X	✓
42	Rottweiler	Male	7 years 7 months	✓	✓
43	Malinois	Male	Unknown	X	✓
44	Rottweiler	Female	5 years 8 months	✓	✓
45	Labrador	Male	6 years 6 months	✓	✓
46	Labrador	Male	6 years 6 months	✓	X
47	Labrador	Male	5 years 9 months	✓	✓
48	Malinois	Male	10 years 10 months	✓	✓
49	Labrador	Male	5 years 8 months	✓	✓
50	Rottweiler	Male	6 years 6 months	✓	X
51	Rottweiler	Male	6 years 6 months	✓	✓
52	Rottweiler	Female	6 years 5 months	✓	✓

GSD, German Shepherd Dog. ✓, sample collected, X, sample not available.

**Table 2:** Overview of the data of the control group.

No.	Breed	Gender	Age
1	Mix breed	Male	8 years 7 months
2	Mix breed	Female	13 years 4 months
3	Mix breed	Female	11 years 7 months
4	Jack Russell Terrier	Female	3 years 9 months
5	Mix breed	Male	4 years 9 months
6	Labrador	Female	14 years 9 months
7	Yorkshire Terrier	Female	3 years 5 months
8	Yorkshire Terrier	Female	12 years 5 months
9	Labrador	Male	13 years 2 months
10	Mix breed	Male	12 years 3 months
11	Mix breed	Female	12 years
12	Pekingese	Female	5 years 4 months
13	Chihuahua	Male	9 years 10 months
14	Mix breed	Female	7 years 4 months
15	Mix breed	Female	4 years 9 months
16	Shetland Sheepdog	Female	6 years 10 months
17	Shetland Sheepdog	Female	10 years
18	Mix breed	Female	4 years 4 months
19	Mix breed	Male	14 years 11 months
20	Mix breed	Female	2 years 7 months

On the day of the initial sample collection, rectal and oropharyngeal swabs were obtained from 39 dogs; The sample collection on the second occasion was conducted in the same way as the first sampling collection. Accordingly, both oropharyngeal and rectal swabs were taken from 47 dogs. Five dogs from the first sampling were not present on-site, while 11 external dogs (No. 1, 8, 9, 20, 21, 25, 26, 34, 37, 41, 43) were tested for the first time. For safety reasons, the oropharyngeal swab of dog No. 4 was omitted at both sampling times. It is noteworthy that one female dog (No. 38) had puppies at the time of the second sampling. After consultation with the responsible veterinarians at the Military Dog Center, it was decided not to test the puppies to avoid subjecting them to stress and to maintain the newly implemented hygiene management, thus preventing potential (re)contamination of the puppies’ kennels. Shortly, the hygiene management included strict separation of the puppies and their mothers from the other dogs in newly built kennels. In addition, the surface materials of the facilities were adapted to ensure optimal and efficient disinfection, mainly with a disinfectant containing potassium peroxymonosulfate effective against parvoviruses. Additionally, the military veterinarians conducted education and training of the staff to optimize hygiene management, while raising awareness among the staff about this issue within the facility.

For the control group, 20 dogs of various ages, breeds, and origins were selected. The main criterion was that the animals did not have diarrhea or any other clinical illness at the time of sampling. Samples were collected from September 2021 to December 2021 in Vienna and Lower Austria. In contrast to the military dogs, only fecal swabs were examined. The owners signed a consent form describing the ongoing study. Additionally, they were provided with instructions for fecal swab collection and storage, as well as a brief questionnaire regarding the medical history of the dogs.

### Retrospective study

In the retrospective part of this study, data from 13 puppies from the Military Dog Center between 2015 and 2021, were analyzed. Only dogs with specific clinical signs (characteristic skin lesions such as blisters, papules, or pustules and/or peracute diarrhea and vomiting—compatible with canine parvovirus enteritis) and positive CBoV-2 PCR findings or contact to PCR-positive littermates, indicating a potential CBoV-2 infection were included in the present study. Overall, seven puppies showed a negative CBoV-2 PCR result but were included due to the characteristic signs and because there were in close contact to CBoV-2 positive littermates. Data and findings were obtained from the Animal Hospital Information System (TIS) of the Clinic for Small Animals, the Institute of Virology, and the Institute of Pathology at the University of Veterinary Medicine Vienna. Documented data regarding the hospitalization were available for eight puppies at the Small Animal Clinic. In four out of these puppies’ pathology reports after necropsy were available. Of Five out of 13 puppies that were not presented at the Clinic for Small Animals, four died, each with an existing pathology report. Virological findings were only available for the fifth puppy. In total, pathology reports were available in eight out of the 13 puppies. Electron microscopic examination, of the intestinal tract was performed in two of these puppies.

### Sample preparation and testing method

One and a half ml (1.5 mL) reaction tubes were filled with 1,000 μL of sterile phosphate-buffered saline (PBS). To obtain sufficient sample material for subsequent molecular biology analysis, the dry swabs from fecal, oropharyngeal, and rectal swabs were rotated in the respective reaction tubes for 60 s in a consistent motion. To avoid contamination between reaction tubes, this step was performed under the safety cabinet. Following this, the reaction tubes were centrifuged at 16000 g for 1 min; after centrifugation, 200 μL of the supernatant were pipetted into a deep 96-well microplate. The reaction tubes with the remaining volumes were then stored as backup samples at −80°C in the freezer. DNA extraction of the samples was performed using the QIAcube HT Instrument (Qiagen, Hilden, Germany) and the QIAamp 96 Virus QIAcube HT Kit (Qiagen, Hilden, Germany) according to the manufacturer’s instructions. 200 μL of each sample were extracted. Sterile PBS was included as a negative control in each extraction round. PCR reaction mixtures were prepared using the FastCycling PCR Kit (Qiagen, Hilden, Germany) following the manufacturer’s instructions. After adding the respective primer pairs (Table 3), amplification was performed in a Mastercycler. For validation, a positive and negative control (extracted PBS) were added to each PCR run. The analysis of PCR products was performed using capillary electrophoresis, QIAxcel Advanced System (Qiagen, Hilden, Germany). The thermocycling conditions for the respective primers used are listed in the Supplementary Tables 1A–C (Data Sheet 1). The entire sample set underwent the following analysis scheme: first, a screening PCR was performed using the primer pairs CBoV-1563-F/CBoV-1782-R. Subsequently, questionable positive samples were verified using two additional CBoV-2 primer pairs (Table 3). A sample that yielded a positive result in any of the alternative primer pairs was interpreted as an overall positive result. Neither CnMV nor CBoV-3 are detected by the primer pairs used.

**Table 3:** Table of the primer pairs used for PCR amplification.

Primer name	Primer sequence	Annealing temperature [°C]
CBoV-1563-F; CBoV-1782-R	5’-GCT GTA CGG ATG TGT GAA-3′; 5′-GTA CAC GTC GTG ATT GGT ACT G-3’	54
CBoV-926-F; CBoV-1993-R	5’-GAG CAA CTC CGT TTG TCT C-3′; 5’-CGT TAG GAG TGC ACT GAA GCT G-3’	52
CBoV-1563-F; CBoV-1925-R	5’-GCT GTA CGG ATG TGT GAA-3′; 5’-GCA TCG AAT CGA GAA GAG C-3’	52

## Results

### Prospective part: first sampling round

Out of the 39 dogs tested the first time in June 2021, 12 (*n* = 12, 31%) were clearly positive, and four (*N* = 4, 10%) were classified as questionably positive. Among the 12 positively tested dogs, CBoV-2-specific nucleic acid was detected in six oropharyngeal swabs and six rectal swabs. Of the four animals classified as questionably positive, half tested positive for CBoV-2 in the oropharyngeal swab and the other half in the rectal swab. During the on-site data collection, it was noted that one dog (Table 4, No. 27) was suffering from watery diarrhea at the time of sampling, and the CBoV-2 screening of the oropharyngeal swab was positive. This dog had shown persistent lesions on the tongue in 2020, and CBoV-2-specific nucleic acid was repeatedly detected from the swab material of these lesions using screening PCR. Later that year, three asymptomatic dogs (No. 32, 38, 44) underwent PCR follow-up examinations using various sample materials (oropharyngeal swab, fecal swab). As part of this study, two of the three dogs (No. 32 and 44) tested positive for CBoV-2, with No. 32 testing positive in the oropharyngeal swab and No. 44 testing positive in the rectal swab.

**Table 4:** Data of dogs from the first sampling round (June 2021) with positive CBoV-2 PCR results.

No.	Breed	Gender	Oropharyngeal	Rectal	Medical history
3	Malinois	male	positive	negative	No notable history
7	Labrador	male	questionable	negative	No notable history, from external breeding (AT)
10	German Shepherd	male	positive	negative	No notable history
12	German Shepherd	male	negative	positive	No notable history
15	Malinois	female	negative	positive	No notable history
19	Tervueren	male	negative	questionable	No notable history
24	Rottweiler	male	negative	positive	No notable history
27	Rottweiler	male	positive	negative	Watery diarrhea, 2020 CBoV positive (symptomatic)
28	Rottweiler	male	negative	positive	No notable history (1 week ago diarrhea)
30	Rottweiler	female	positive	negative	No notable history
32	Rottweiler	female	positive	negative	2020 CBoV positive (asymptomatic)
39	Malinois	male	positive	negative	No notable history
44	Rottweiler	female	negative	positive	No notable history, 2020 CBoV positive (asymptomatic)
45	Labrador	male	questionable	negative	No notable history
47	Labrador	male	negative	questionable	No notable history
48	Malinois	male	negative	positive	No notable history

During the second round of testing 2 months later, one (2%) dog (No. 15) tested positive in the oropharyngeal swab, and two others (4%; No. 7, 45) tested with a questionably positive result in the oropharyngeal swab.

### Second sampling round

Among the 47 dogs tested at the end of August 2021 for the second round of testing, one (2%) female dog tested positive in the oropharyngeal swab, and two others (4%) tested with a questionably positive result in the oropharyngeal swab. None of the dogs exhibited clinical abnormalities at the time of sampling.

### Control group

The PCR testing for CBoV-2 in the entire control group returned negative results.

### Retrospective study

For the evaluation of retrospective cases, data from a total of 13 Rottweiler puppies (6 female, 7 male) from the military dog breeding unit were evaluated. The affected puppies from litters A to D, as well as two individual cases without litter affiliation (No. 3y and 10z), were housed in kennels from birth and had no contact with other dogs outside the premises. The median age of the puppies was 2.6 months old (range 2–4 months) at the onset of clinical signs. The affected dogs consistently exhibited uniform signs, consisting of characteristic skin lesions and gastrointestinal signs. CBoV-2 was detected by conventional PCR from fecal swabs, gastrointestinal tract, and/or skin samples in six out of 13 of the puppies. Due to the similarity of clinical signs among the puppies within each litter, the seven negative-tested puppies were also included in the retrospective study. Given the small number of animals, a purely descriptive analysis of the documented cases and no statistical analysis was performed. Five of the six dogs tested positive (No. 1A, 4B, 11D, 12D, and 13D) died or were euthanized. The median duration from onset of clinical signs to death was 35 days (range 14 to 91 days). Dogs No. 1A and 12D died 14 days after the first sign appeared; No. 4B died 35 days after onset of clinical signs; No. 11D died 91 days after onset of signs. This puppy relapsed twice during this period. Puppy No. 13D died 60 days after it had been presented with clinical signs. Except for disseminated skin lesions, this dog showed no other relevant signs until it died. Of the seven puppies that tested negative, three (No. 2A, 3y, and 4B) died. Puppy No. 3y died within 21 days after onset of clinical signs; detailed courses were not ascertainable for dogs No. 2A and 4B due to lack of data. An overview of the data can be found in Table 5.

**Table 5:** Data overview of the dogs of the retrospective part of the study.

No.	Litter	Age	Gender	CBoV-2 PCR	Hospitalization	Pathology report	Pathology report suspicious for CBoV	Outcome
1	A	2.6 months	f	+	no	yes	Yes	Deceased
2		2.6 months	m	−	no	yes	Yes	Deceased
3y		3 months	m	−	yes	yes	No	Deceased
4	B	3 months	m	+	yes	yes	Yes	Euthanasia
5		2 months	f	−	no	yes	Yes	Deceased
6	C	2 months	m	−	yes	no		Recovered
7		2 months	f	−	yes	no		Recovered
8		2 months	f	−	yes	no		Recovered
9		2 months	m	−	yes	no		Recovered
10z		3 months	m	+	no	no		Recovered
11	D	4 months	f	+	yes	yes	Yes	Euthanasia
12		4 months	m	+	yes	yes	Yes	Deceased
13		4 months	f	+	yes	yes	Yes	Deceased

All 13 dogs exhibited skin lesions at different body sites, presenting as blisters, papules, or pustules. Other commonly documented signs, in descending order, included: diarrhea (83%), vomiting (77%), as well as respiratory (15%) and neurologic (8%) abnormalities. Initially, the majority (12/13) of the puppies presented to the clinic due to hemorrhagic-watery diarrhea, frequently accompanied by vomiting. The attending veterinarians described the diarrhea as compatible with CPV-2 infection. The intensity and quality of diarrhea and vomiting were not individually recorded and therefore cannot be adequately assessed retrospectively. In addition to these two initial signs, the animals also exhibited nonspecific signs such as apathy, anorexia, abdominal pain, and mildly elevated rectal temperature.

**Figure 1:**
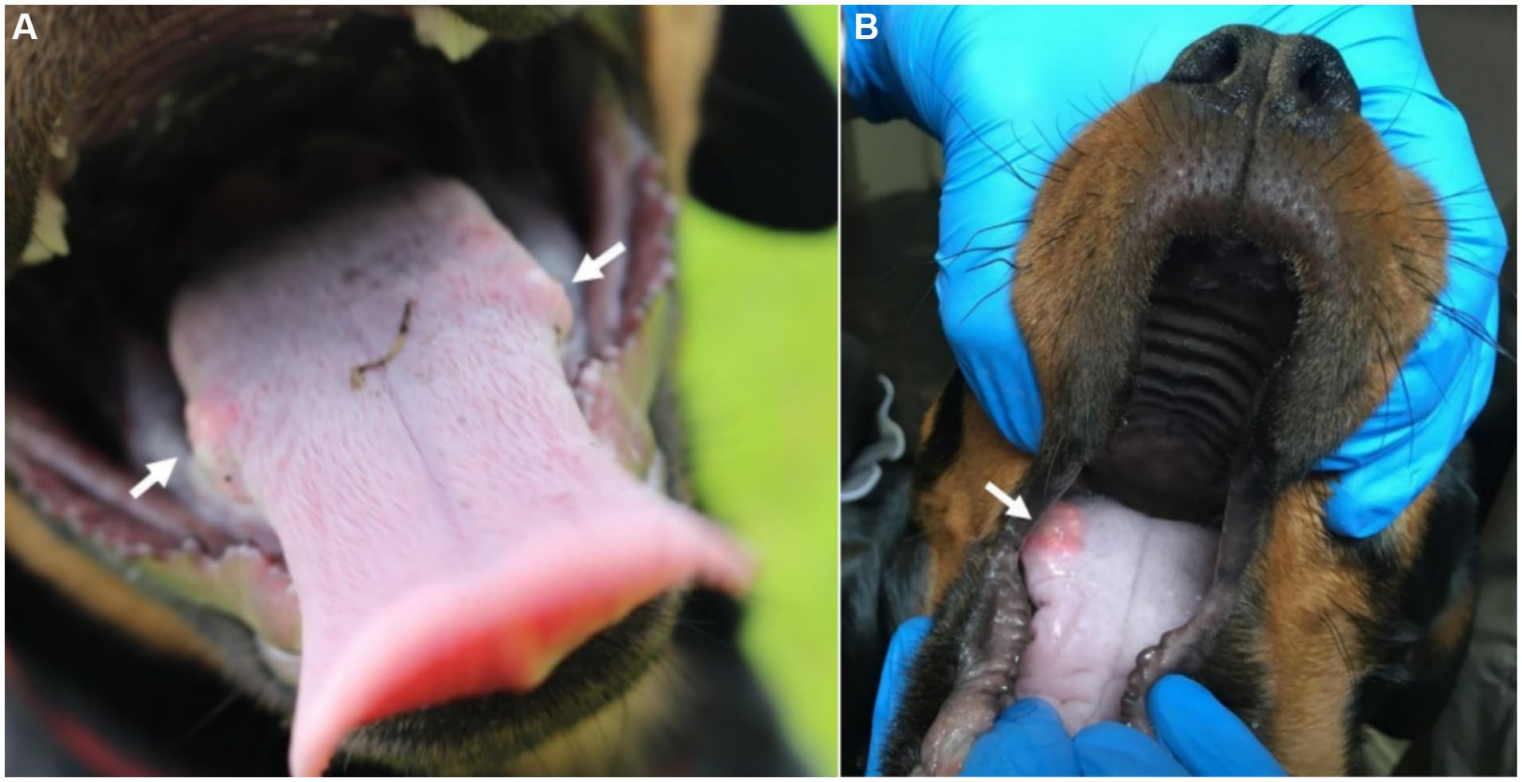
**(A,B)** Mucosa lesions from Puppy Nr 10z. **(A)** Persistent lesion at the posterior third of the tongue (arrow indicating the tongue lesion). **(B)** Bilateral lesion on the tongue (arrow indicating the tongue lesion). The lesions depicted in this figure are associated with PCR detection of CBoV-2 DNA. However, the causal relationship between these lesions and CBoV-2 remains unproven and should be interpreted with caution.

**Figure 2:**
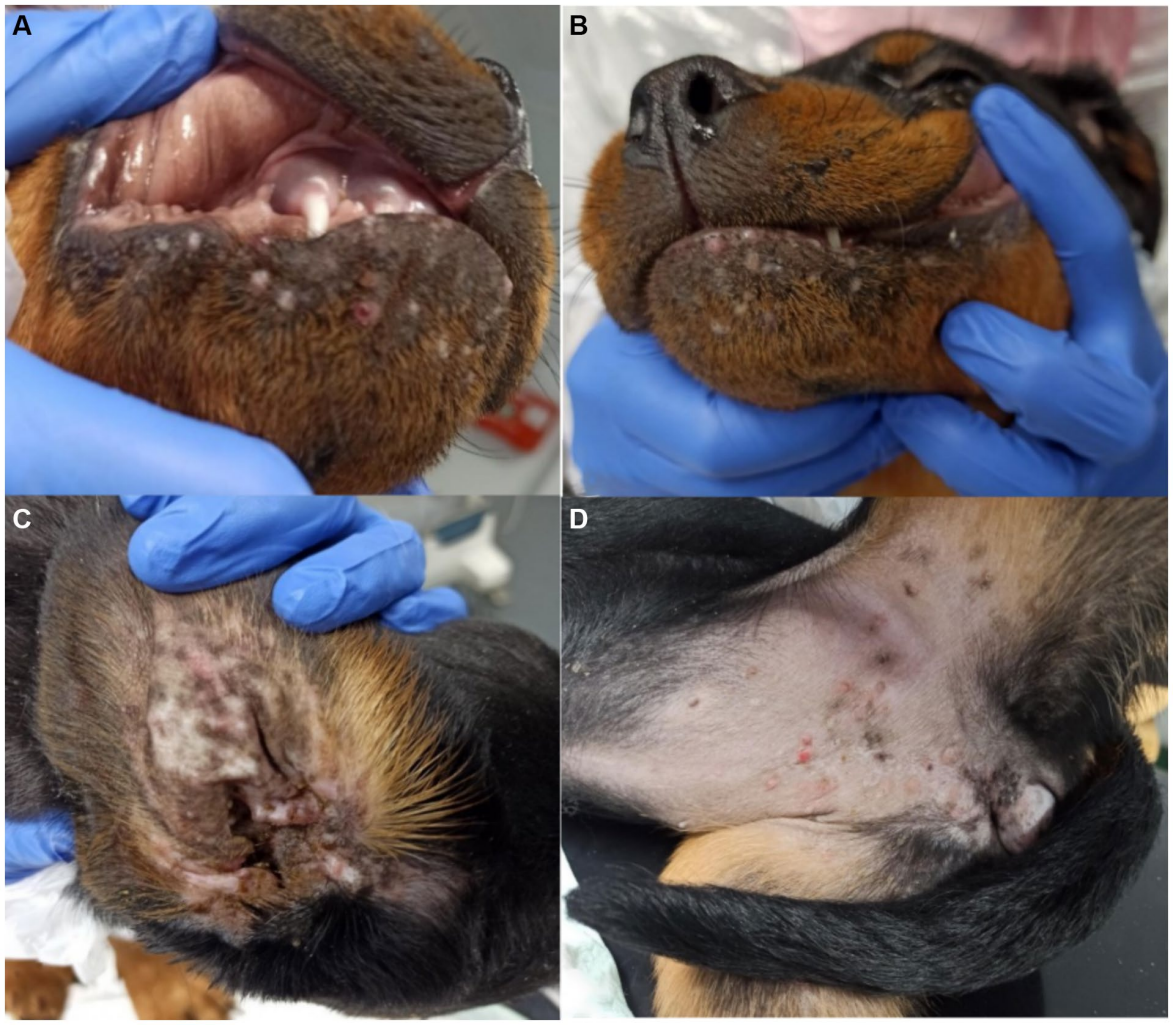
**(A–D)** Pictures of skin lesions from Puppy Nr 11D. **(A,B)** perioral pustules and papules. **(C)** Lesions on the inner side of the ear pinna. **(D)** Skin lesions pustules and papules on the ventral abdomen. The lesions depicted in this figure are associated with PCR detection of CBoV-2 DNA. However, the causal relationship between these lesions and CBoV-2 remains unproven and should be interpreted with caution.

CBoV was detected in feces and intestine from six puppies (Nr. 1A, 4B, 10z, 11D, 12D, 13D), while the testing of the remaining dogs yielded negative results. Among the eight dogs with pathology reports, no uniform pattern was observed. However, the findings in seven puppies suggested that primary changes in the gastrointestinal tract were decisive for the death of the puppies. In 3/8 puppies, there was evidence of intestinal intussusception, interpreted as the cause of death (Nr. 2A, 3y, 12D). In two other puppies (Nr. 1A, 4B), enteritis was diagnosed during pathological examination, with one of these dogs also having pancreatitis (Nr. 4B). Two dogs (Nr. 5B, 11D) each showed mild to severe purulent-necrotizing hepatitis, with puppy Nr. 5B also having intestinal fibrosis, and puppy Nr. 11D having mild multifocal ulcerative-purulent colitis. Puppy Nr. 13D had chronic inflammation of the cecal wall, but the primary cause of death was chronic purulent myocarditis in combination with fibrinous epicarditis and pericarditis and left ventricular hypertrophy. Histologically, the changes in the caecum wall resembled those of the heart. The skin lesions were macroscopically described as papules, blisters, or pustules. The localization of the skin lesions was documented in all puppies on the inner side of the ear pinnae, and additionally on the ventral abdomen in six puppies (Nr. 1A, 2B, 6C, 7C, 8C, 9C). One dog (Nr. 4B) had pustules and erythema on the inner surfaces of the ear pinnae, as well as pustules on the carpal joints, axillae, and ventral neck. Mucous membranes and mucocutaneous junctions were affected in four puppies as follows: Nr. 10z (according to history, multiple pustules in the oral cavity; Figure 1), Nr. 3y (persistent skin lesions on the tongue mucosa), Nr. 11D (multiple blisters on the oral mucosa; Figure 2), Nr. 13D (multiple nodules and crusts on the vulva). Three dogs (Nr. 4B, 11D, 13D) had pruritic pustules. Bacteriological examinations (*n* = 3) of the affected skin areas revealed colonization with *Staphylococcus pseudintermedius*, *Corynebacterium* spp., and *Escherichia coli*. Testing for dermatophytes was negative in one dog. Histopathological examination of three skin biopsies was performed on dog Nr. 4B, revealing superficial pyoderma and cocci in the infundibulum. Results of skin biopsies in puppies revealed comparable histopathological findings that showed purulent dermatitis with bacterial infiltration. While some animals showed improvement of skin lesions during hospitalization, an otherwise clinically normal puppy (Nr. 10z) had persisting tongue lesions over several months (Figures 1A,B). CBoV-2 nucleic acid was detected by screening PCR from tongue swab material. Five other puppies from the same litter (total of 8 puppies) had pustular lesions that resolved over an unspecified period. CBoV-2 nucleic acid was detected by PCR from skin lesion swabs in three dogs (Nr. 10z, 11D, 13D); in one dog (Nr. 12D), the skin sample was found positive along with intestinal tissue in a pooled sample. The remaining puppies’ skin material was not examined by PCR. The skin lesions (perioral, inner ear, and ventral abdomen) of puppy Nr. 11D are shown in Figures 2A–D. Two puppies (puppies Nr. 4B and 5B) from the same litter were affected by respiratory signs. During their hospitalization, mucopurulent discharge was observed in these puppies. However, upon necropsy, there were no significant macroscopic or histopathological changes in the lungs. Puppy Nr. 4B also exhibited neurological signs. During neurological examination, the puppy showed lateralized ataxia, increased yawning, self-mutilation on the hind limb paws, and intermittent tremors in the head and neck. Examination of the cerebrospinal fluid revealed a pleocytosis dominated by lymphocytes. The conducted CDV rapid test (FASTest® DISTEMPER Strip, MEGACOR Diagnostik GmbH, Hörbranz, Austria) yielded negative results. Post-mortem testing of urine, whole blood, as well as organ samples from the skin and brain using screening PCR for CBoV-2 also returned negative results for this puppy. Three PCR tests performed ante mortem from the puppy’s fecal swab yielded positive results. The necropsy revealed a pyogranulomatous meningoencephalitis of unknown etiology. The hematological and biochemical parameter values of the hospitalized patients are displayed in the Table 6.

**Table 6:** Table displaying hematological and biochemical parameters in hospitalized patients.

	Animal number							
Parameter	3y	4B	8C	11D	12D	13D	Reference range	SI unit
Erythrocytes (10^^6^/μL)	3.61	5.10	4.19	5.2	5.59	5.41	5.50–8.00	10^12^/L
Hemoglobin (g/dL)	6.2	10.3	10.3	10.1	11.4	11.5	12.0–18.0	mmol/L
Hematocrit (%)	18.9	31.7	33.2	31.7	35.0	35.4	37.0–55.0	L/L
Platelets (10^^3^/μL)	956	438	484	615	506	584	150–500	10^9^/L
Leukocytes (/μL)	19,150	11,050	26,400	38,260	32,660	17,490	6,000–15,000	10^6^/L
Band Neutros. (/μL)	383	0	1848	0	0	0	<500	10^6^/L
Segm. Neutros. (/μL)	14,745	7,436	18,216	30,914	26,781	12,890	3,300–12,500	10^6^/L
Lymphocytes (/μL)	1723	1,359	4,488	3,213	1959	2011	780–4,500	10^6^/L
Monocytes (/μL)	2,298	872	1,056	2,946	3,266	1,311	<500	10^6^/L
Eosinophils (/μL)	0.02	1,292	792	918	653	1,171	<800	10^6^/L
GLU (mg/dL)	102	95	NA	112	66	126	55–90	mmol/L
BUN (mg/dL)	15.9	18.3		34.3	10.9	NA	20.0–40.0	mmol/L
CREA (mg/dL)	0.30	0.40		0.40	0.20	0.50	0.40–1.20	μmol/L
TP (g/dL)	3.61	5.19		3.95	2.55	5.06	6.00–7.50	g/L
ALB (g/dL)	1.46	2.69		2.07	1.16	3.15	2.58–4.73	g/L
ALP (U/L)	128	257		207	105	NA	<130	μkat/L
ALT (U/L)	18	89		11	8	17	<80	μkat/L
CRP (mg/dL)	59.2	34.5		51.4	104.0	11.8	<35.0	mg/L

Neutros, neutrophils; Segm. Neutros, segmented neutrophils; GLU, Glucose; BUN, blood urea nitrogen; CREA, Creatinine; TP, Total Protein; Alb, Albumin; ALP, Alkaline Phosphatase; ALT, Alanine Aminotransferase; CRP, C-reactive Protein; NA, not done.

The specimens from all 13 dogs underwent conventional screening PCR for CBoV-2. Principal sample materials included fecal swabs, along with skin tissue or swabs from modified skin and mucosal regions, as well as organ specimens procured during autopsies. In one deceased patient (No. 12D), after pathological scrutiny, CBoV-2-specific nucleic acid was discerned via PCR in various organs (comprising liver, kidney, spleen, brain, skin, small intestine, and lung tissue). Regarding puppy 2A, the screening PCR yielded negative results for intestinal samples; nevertheless, virus particles exhibiting characteristic parvovirus morphology were observed during electron microscopic evaluation of the intestine. Immunohistochemistry excluded CPV-2 and CDV as causative agents. Furthermore, investigations aimed at identifying other differentially relevant viruses associated with gastroenteritis were instigated in select puppies. For three animals (No. 4B, 10z, 11D) manifesting dermatological indications, a canine herpes virus 1 (CHV-1) assay was conducted, yielding negative outcomes in all instances. The above-mentioned results and an elucidation of the methodologies employed to exclude differentially relevant viruses (inclusive of utilized sample materials and employed detection techniques) are delineated in Table 7 and the pathology diagnosis is given in Table 7. In 46% (6/13) of cases, a co-infection with coccidia (*Cystoisospora ohioensis*) and/or *Giardia* spp. was observed during parasitological fecal examination. The parasitic infestation was uniformly classified as severe. Four puppies (C litter) had a monoinfection with *Cystoisospora ohioensis*. *Giardia* spp. was detected in one puppy (Nr. 12D). One puppy (Nr. 4B) was infected with both *Cystoisospora ohioensis* and *Giardia* spp.

**Table 7:** Table of CBoV-2 results, results of other viral testing and final pathology results.

Nr.	CBoV-2 PCR	Additional viruses excluded	Material	Method	Pathology diagnosis
1A	+	CDV, CPV-2	Intestinal tissue	IHC	CBoV-associated enteritis
2A	−	CDV, CPV-2	Intestinal tissue	IHC	small intestinal intussusception
3y	−	CPV-2	Fecal swab	Snap-Test, PCR	necrotic invagination of jejunum and ileum
4B	+	CDV, CECoV, CPV-2, CHV-1	CSF, Fecal swab	Snap-Test, PCR	pyogranulomatous meningoencephalitis, chronic enteritis, pancreatitis
5B	−	CECoV, CPV-2	Intestinal tissue	PCR	severe suppurative hepatitis, intestinal fibrosis (duodenum and colon)
6C	−	NA			
7C	−	NA			
8C	−	CPV-2	Fecal swab	PCR	
9C	−	NA			
10z	+	CHV-1, Pan-Pox	Tongue swab	PCR	
11D	+	CHV-1, CPV-2	Skin swab, fecal swab	PCR	moderate multifocal suppurative hepatitis, moderate multifocal ulcerative suppurative colitis
12D	+	CPV-2	Fecal swab	Snap-Test, PCR	small intestinal intussusception with hemorrhagic infarction, necrosis of intestinal wall with rupture and consecutive fibrinous suppurative peritonitis
13D	+	NA			chronic suppurative myocarditis, fibrinous epicarditis and pericarditis, left ventricular hypertrophy resulting in heart failure, chronic inflammation of cecal wall

IFAT, Immuno Fluorescence Antibody Test; IHC, Immunohistochemistry; NA, not applicable (no information provided); Snap-Test, Snap Parvo-Test (IDEXX GmbH, Kornwestheim, Germany); PCR, Polymerase Chain Reaction; CBoV-2, Canine bocavirus 2; CDV, Canine Distemper Virus; CECoV, Canine Enteric Coronavirus; CHV-1, Canine Herpesvirus 1; CPV-2, Canine Parvovirus 2; Pan-Pox, Poxviruses.

## Discussion

In the present study we describe the first evidence of CBoV-2 in Austria. To date, the canine bocavirus has only been sporadically documented in the scientific literature (3–7, 9, 10). Especially in the context of cohort investigation, only a few review articles exist (3). Regarding canine bocaviruses, Koch’s postulates have not been fulfilled due to the lack of *in vivo* and *in vitro* experiments. Therefore, clinical signs or pathological changes suspected to or observed in association with CBoV-2 cannot be conclusively assumed to be causative for the present clinical manifestations. Much more, it can be assumed that CBoV-2 acts opportunistically and/or synergistically with other pathogens (4).

In the prospective part of the present study, the first sampling round revealed a prevalence of CBoV-2 in 31% of the dogs in the examined population, while only 2% tested positive during the subsequent round of sampling. Among all of the tested canines, either the oropharyngeal (*N* = 7) or rectal swab (*N* = 6) yielded positive results; however, no dog tested positive in both swabs. This discrepancy may be attributed to the hypothesis that dogs testing positive in the oropharyngeal swab may have acquired the virus orofecally or orally from contaminated surfaces. An alternative explanation for the molecular detection of CBoV in the oropharynx could be the virus’s tropism to tonsils; however, empirical evidence regarding this phenomenon, along with insights into the pathogenesis and replication mechanism, remains scarce. Furthermore, it is possible that it is DNA evidence of different CBoV-2 variants, which have different cell tropisms. Further investigations, such as sequence analyses of the PCR products, are necessary to solve this puzzle. Studies reporting the presence of human bocavirus (HBoV) within tonsils offer credence to this hypothesis (13). Moreover, the positive dogs exhibited no clinical signs and likely harbored lower viral loads compared to individuals showing clinical signs. Nevertheless, quantitative PCR analysis would be necessary to validate this assumption. The highly contagious canine parvovirus type 2 (CPV-2), is the most important virus of the *Parvoviridae* family in small animal medicine (14, 15). It can escalate to systemic inflammatory response syndrome (SIRS), sepsis, shock, and/or multiple organ failure, with a mortality rate of up to 91%, especially if left untreated (16–18). Additionally, CPV-2 infections may rarely cause neurological or cutaneous signs. Transplacental infection from unvaccinated or inadequately vaccinated female dogs, or infection in puppies under 6 weeks of age can result in CPV-2-associated myocarditis, which leads to more guarded prognosis (15, 19). Numerous studies cover the genetic and molecular particularities of CPV-2. Unlike to CPV-2, dedicated investigations into the enhanced environmental persistence of CBoV-2 are lacking, primarily due to the absence of a functional cell culture system. Solely one dog (No. 27) from the prospective cohort displayed clinical manifestations (mild diarrhea) on the first sampling day. Although this dog tested positive solely in the oropharyngeal swab during the first assessment, it tested negative during the subsequent evaluation. This dog (also included in the retrospective part; No. 10z) had previously exhibited persistent lesions on the tongue or oral mucosa in 2020, from which CBoV-2 was recurrently detected via PCR. Importantly, this dog was the sole participant of the study showing alterations only in the oral mucosa, distinguishing it from the remaining 12 dogs. This dog might represent a persistent CBoV-2 carrier; however, conclusive evidence supporting this assertion is currently unavailable.

The substantial disparity in virus detection frequencies between the two examination days could potentially be attributed to the fact that the first sampling was close to the clinical cases in the cohort and the possibly increased virus circulation at that period. Also, it can be assumed that at the time of the second sampling the development of antibodies could have offered a protection to the cohort. Additionally, a beneficial role of the implementation of newly devised hygiene protocols at the military dog center can be presumed. Following antecedent puppy losses, a novel hygiene regimen was collaboratively developed by the authors and enacted in early summer 2021. Since the initiation of the enhanced hygiene measures, no further puppy mortalities have been reported until now (June 2024), suggesting a correlation with the heightened hygiene standards. An alternate explanation for the disparate frequency rates might lie in the intrinsic characteristics of the virus itself, potentially manifesting in seasonal fluctuations. A notable instance of seasonally contingent prevalence in viral diseases is observed with CPV-2 (peaking in May–June) (20). Analogous discussions concerning the seasonal occurrence of HBoV are also extant (13). However, literature pertaining to the seasonal prevalence of CBoV-2 remains scant. In total, six dogs (4 in the first examination, 2 in the second examination) were provisionally classified as positive. The questionable PCR results can be explained by the heterogeneity of the circulating CboV-2 sequence in the population. The results of the control group (*N* = 20) were consistently negative. However, the limited number of examined dogs is a limiting factor. Nevertheless, due to the numerous positive dogs in the military dog population, it can be assumed that there is increased infection pressure, as many dogs are kept in close quarters. It should be noted, however, that different sample materials were tested. To minimize stress on the animals and potential injuries during sample collection, the control group animals and their owners were only sampled with swabs of freshly deposited feces. Owners were instructed on how to properly collect swab samples of their animals’ freshly deposited feces to avoid contamination.

A purely descriptive data analysis was performed on the retrospectively evaluated cases. A detailed description of the disease progression, therapeutic intervention, etc., was omitted due to the lack of comparability stemming from the heterogeneity of the data. Additionally, some puppies were included even if they yielded negative CBoV-2 results. Nevertheless, the samples of these individuals were incorporated into the study due to the similarity of exhibited signs or their respective litter affiliation. This decision was made as false-negative results from virological detection methods could not be definitively ruled out. Moreover, negative PCR might have been a results of the virus not being eliminated at the time of sampling.

In the litter D (No. 11D–13D), all three puppies tested positive for CBoV-2 by PCR. In two separate litters, one puppy tested positive while the other tested negative, despite presenting with very similar clinical manifestations, both of which resulted in fatalities. It is plausible to speculate that the negatively tested dogs may have simply not been shedding the virus at the time of testing due to intermittent excretion pattern of parvoviruses in dogs. While CBoV-2-specific nucleic acid was detected in the fecal samples of puppy No. 1A, it was not detected in No. 2A. However, electron microscopy of the intestines of puppy No. 2A revealed numerous virus particles with a typical parvoviral morphology, coupled with the exclusion of CPV-2 infection, suggesting that puppy No. 2A was also infected with CBoV-2, although infection with other canine parvoviruses that were not tested cannot be ruled out. In a virological examination, deceased puppy No. 11D exhibited separate PCR detection of CBoV in various organs (spleen, kidney, liver, skin, brain, lungs, and small intestine). Histological examination revealed multifocal micro abscesses in the liver and karyorrhexis of numerous follicular lymphocytes in the spleen. Regardless of the positive CBoV-2 PCR, no histological abnormalities were observed in the kidney, lungs, small intestine, or brain. The presence of viral nucleic acid in these organs could be attributed to a preceding viremia, a phenomenon documented in the literature, such as the detection of CBoV-2 in the liver or brain without histopathological changes (5, 6). The puppies of the C litter (No. 6C–9C) exhibited signs (vomiting, diarrhea, skin lesions) potentially associated with clinically manifest CBoV-2 infection but consistently tested negative for CBoV-2. The entire C litter was deemed to have recovered after a short illness duration, with no recurrence of clinical symptoms. One theory for the negative results could be a low viral load, which may influence the disease outcome, although publications on this topic are scarce. The establishment of quantitative PCR (qPCR) could potentially aid in detecting dogs with low viral loads in the future. In general, it appears to be difficult to detect all Austrian CBoV-2 variants with a single PCR assay. Attempts to establish a qPCR have so far failed because it worked well for the cloned standard and some of the positive samples, but not for other demonstrably positive samples (data not shown).

The signs described in the literature (diarrhea, vomiting, respiratory, and neurological abnormalities) were also observed in the dogs included in the retrospective cases of the military dog population (3, 4). Moreover, a study evaluated brain samples from neurologically diseased and deceased dogs (*n* = 107) for CnMV, CBoV-2, and CBov-3 by PCR. Affected dogs exhibited signs such as seizures, behavioral changes, gait disturbances, disorientation, weakness, balance issues, and/or cervical spine pain. Among the CBoV-positive samples (prevalence 14%), only CBoV-2 was identified, predominantly in young animals (60%) (6). Ten of the CBoV-2-positive dogs presented epileptic seizures. Other viruses commonly associated with neurological signs in dogs [e.g., rabies, canine distemper virus (CDV), canine adenovirus, CPV-2] were not detected in the examined samples. The authors also conducted *in situ* hybridization and transmission electron microscopy of the tissue to verify the localization of CBoV-2, which largely correlated with the positive PCR results. Consequently, the authors hypothesize a potential tropism of CBoV-2 for the brain. They further emphasize that several studies suggest bocaviruses may trigger viremia, subsequently crossing the blood–brain barrier and potentially inducing lesions in the central nervous system (6). Subsequent *in situ* hybridization confirmed porcine bocavirus (PBoV) as a possible causal agent of observed histological brain lesions (21). Furthermore, human bocavirus (HBoV) has been isolated from central nervous system (CNS) material of patients suffering from encephalitis of unknown etiology. In cerebrospinal fluid samples from children with encephalitis, HBoV was detected in 3–15% of cases (22, 23). Additionally, all affected puppies included in this study exhibited comparable skin changes in the form of blisters, papules, and pustules, predominantly observed in specific body regions (especially the inner ear pinnae and ventral abdomen). Such skin lesions have not been previously described in association with CBoV-2 infection in dogs. There are only two publications reporting HBoV-positive children with possible HBoV-associated skin lesions, presenting as maculopapular erythema on the chest and face, as well as an exanthema subitum in one child (mainly affecting the trunk) (24, 25). Similarly, individual case reports of CPV-2 infections have described comparable skin lesions, such as ulcers on paw pads and tongue, mucosal erythema, blisters in the oral cavity, and disseminated erythema and alopecia throughout the body (26). While the skin lesions described in this study clinically differed from those associated with HBoV, there are certain similarities with the localization and nature of dermatological manifestations caused by CPV-2, particularly involving mucosal surfaces.

Our study has some limitations. While PCR is a powerful tool for detecting CBoV-2 DNA, it does not distinguish between active infection and the presence of non-infectious viral particles or remnants from past exposure. Therefore, the presence of viral DNA should be interpreted with caution, and complementary diagnostic methods (like viral culture and serology) are necessary to confirm active infection. Unfortunately, these tests are not available at the time and therefore, were not performed. One limitation of the present study is the small number of skin samples tested from the dogs to establish a direct link between skin lesions and CBoV-2 infection. Only three dogs (No. 10z, 11D, 13D) underwent conventional PCR testing for CBoV-2 on mucosal or skin samples, and an additional dog’s (No. 12D) skin material was pooled with intestinal tissue for PCR analysis. While all four results yielded positive PCR outcomes, the association with CBoV-2 cannot be definitively established due to inadequate examination for other potential causes. However, two of these dogs (No. 10z and 11D) also tested negative for CHV-1 via PCR, which is a major differential diagnosis for skin lesions, suggesting a possible CBoV-2-associated dermatological manifestation in these cases (19, 27). Another CBoV-2 positive puppy (No. 4B; positive PCR from fecal swab) tested negative for CHV-1 via serological examination (IFAT). Nevertheless, the consistent affected skin areas and types of skin lesions observed in the 13 puppies provide evidence that CBoV-2 may be the causative agent. In the Case of puppy 2A, considering the medical history, the findings, and the demise of a CBoV-2 positive patient from the same litter exhibiting comparable signs (No. 1A), an association with CBoV-2 infection is only postulated. CBoV-2 detection using *in-situ* hybridization reported in a previous study also did not yield reliable positive results, presumably due to nucleotide differences at the probe binding site (data not shown) (28). The association between CBoV-2 and the observed lesions in this study is based on PCR detection of viral DNA. However, without experimental evidence or further histopathological confirmation, it cannot be confirmed that these lesions are directly caused by CBoV-2 or are coincidental findings. The establishment of additional diagnostic techniques (such as immunohistochemistry) would be essential for further confirmation of potential CBoV-2-associated dermatological manifestations and future studies should aim to establish a causal relationship through controlled experimental infections and to enhance our understanding of CBoV-2’s role in canine health.

The initial blood findings including a total of 6 puppies were included in the retrospective part of the study. Of these six puppies, five tested positive for CBoV-2. Although one dog tested negative for CBoV-2 via PCR, it was included in the study based on characteristic signs and negative CPV-2 PCR results. All six puppies exhibited mild to moderate anemia, likely related to their young age. Interestingly, five puppies showed moderate leukocytosis, with all eight puppies displaying monocytosis and 5 out of 6 puppies showing neutrophilia, contrasting with CPV-2 infection, which typically results in significant neutropenia. Half of the puppies exhibited eosinophilia, with only one puppy testing positive for *Cystoisospora ohioensis* and *Giardia* spp., which could potentially explain the eosinophilia. Four dogs displayed hyperglycemia, five hypoproteinemia, and three hypoalbuminemia, likely associated with diarrhea. Two exhibited elevated alkaline phosphatase levels probably due to growth-related factors. The interpretative value of the blood findings is limited due to the small number of dogs with available blood results, and thus, comparisons with other studies cannot be made. It is also challenging to determine from the available data whether CBoV-2 was causative for the described hematological changes in the puppies or if bacterial secondary infections were influential.

Another limitation of this study is the diagnostic approach used to exclude differentially relevant viral gastroenteritis. While the tests conducted yielded negative results, there was no uniform testing regimen in the 13 puppies due to the retrospective nature of the study. For instance, CPV-2 was mostly ruled out (8/13) using various methods (antigen rapid test, PCR), but data were lacking for four puppies. Additionally, no extensive molecular testing was performed to exclude canine adenovirus. However, no indications of CAV infection were found during the histopathological examinations (e.g., inclusion bodies). Similarly, PCR testing for canine enteric coronavirus (CECoV) was only conducted for two out of the 13 puppies. As mentioned earlier regarding the dermatological changes, there was also no uniform testing scheme for epitheliotropic viruses. The lesions observed in this cohort may be influenced by a variety of factors, including co-infections, environmental stressors, or non-specific inflammatory responses. While CBoV-2 DNA was detected in these cases, it is important to consider alternative explanations and the potential for false positives due to environmental contamination. Therefore, it would be beneficial for future studies to implement a standardized diagnostic approach for affected puppies in the population. None of the dogs with available pathological findings exhibited similarities to CPV-2 enteritis [e.g., necrosis of crypt epithelium, shortened and atrophied villi] (15).

In conclusion, this study demonstrates that up to one-third of dogs in a specific population tested positive for CBoV-2, with some of these dogs also presenting clinical signs. However, it remains unclear whether CBoV-2 was causative for the observed clinical symptoms or acted synergistically with bacterial/parasitic gastroenteritis. While our study provides initial insights into the presence of CBoV-2 in a specific canine population, the findings should be interpreted with caution. The detection of CBoV-2 DNA alone does not confirm an active infection or establish a causal link with the observed clinical signs. Further studies are essential to address whether CBoV-2 infections pose a threat to young animals within specific dog populations or within the general dog population. When characteristic symptoms associated with CBoV-2 (especially in puppies or young dogs) are present, differential diagnosis should consider CBoV-2. Confirmation through a positive CBoV-2 result may prompt evaluation and adjustment of hygiene standards, as evidenced by the observed absence of diseased puppies in the described dog population following the implementation of adequate hygiene management adjustments. Further diagnostic testing like viral culture and *in situ* hybridization are crucial to support diagnosis and causality with the clinical signs. Additionally, the establishment of a method for detecting antibodies against CBoV-2 would be valuable, providing insights into the prevalence of CBoV-2 in a dog population. Furthermore, the validation of qPCR would aid in interpreting the significance of pathogen detection. Despite numerous limitations, this study offers insight into an under-documented virus, its potential clinical manifestations, and its role in the context of population issues.

## Data Availability

The original contributions presented in the study are included in the article/Supplementary material, further inquiries can be directed to the corresponding author.
