# Supplementary material for: Prevalence and significance of a canine bocavirus-2 outbreak in a cohort of military dogs in Austria
Source: Front Vet Sci. 2024 Sep 5;11:1461136. doi: 10.3389/fvets.2024.1461136 (PMC11410759; doi:10.3389/fvets.2024.1461136)
Supplement: Supplementary file 1 [file Data_Sheet_1.PDF]

**Step**	**[°C]**	**Duration**	**Number of Cycles**
Initial Denaturation	95	5 Min	1x
Denaturation	96	5 Sec	
Annealing	54	5 Sec	45x
Elongation	68	9 Sec	
Final Elongation	72	1 Min	1x

Supplementary Table 1a. Thermocycling Conditions for Primers CBoV-1563-F/1782-R

**Step**	**[°C]**	**Duration**	**Number of Cycles**
Initial Denaturation	95	5 Min	1x
Denaturation	96	5 Sec	
Annealing	52	5 Sec	49x
Elongation	68	40 Sec	
Final Elongation	72	1 Min	1x

Supplementary Table 1b. Thermocycling Conditions for Primers CBoV-926-F/1993-R

**Step**	**[°C]**	**Duration**	**Number of Cycles**
Initial Denaturation	95	5 Min	1x
Denaturation	96	5 Sec	
Annealing	52	5 Sec	50x
Elongation	68	12 Sec	
Final Elongation	72	1 Min	1x

Supplementary Table 1c. Thermocycling Conditions for Primers CBoV-1563-F/1925-R
